# Biophysical, Biochemical, and Cell Based Approaches Used to Decipher the Role of Carbonic Anhydrases in Cancer and to Evaluate the Potency of Targeted Inhibitors

**DOI:** 10.1155/2018/2906519

**Published:** 2018-07-16

**Authors:** Mam Y. Mboge, Anusha Kota, Robert McKenna, Susan C. Frost

**Affiliations:** Department of Biochemistry and Molecular Biology, University of Florida, College of Medicine, P.O. Box 100245, Gainesville, FL 32610, USA

## Abstract

Carbonic anhydrases (CAs) are thought to be important for regulating pH in the tumor microenvironment. A few of the CA isoforms are upregulated in cancer cells, with only limited expression in normal cells. For these reasons, there is interest in developing inhibitors that target these tumor-associated CA isoforms, with increased efficacy but limited nonspecific cytotoxicity. Here we present some of the biophysical, biochemical, and cell based techniques and approaches that can be used to evaluate the potency of CA targeted inhibitors and decipher the role of CAs in tumorigenesis, cancer progression, and metastatic processes. These techniques include esterase activity assays, stop flow kinetics, and mass inlet mass spectroscopy (MIMS), all of which measure enzymatic activity of purified protein, in the presence or absence of inhibitors. Also discussed is the application of X-ray crystallography and Cryo-EM as well as other structure-based techniques and thermal shift assays to the studies of CA structure and function. Further, large-scale genomic and proteomic analytical methods, as well as cell based techniques like those that measure cell growth, apoptosis, clonogenicity, and cell migration and invasion, are discussed. We conclude by reviewing approaches that test the metastatic potential of CAs and how the aforementioned techniques have contributed to the field of CA cancer research.

## 1. Introduction 

The role of CAs in different human disease states has been studied for many decades [1–3]. These studies have led to the development of targeted therapy against CAs that are currently in clinical use for the treatment of diseases such as glaucoma, altitude sickness, and epileptic seizures. CA targeting inhibitors are now being tested in preclinical models for the treatment of diabetes and obesity, while others are currently in clinical trials as cancer therapies [4–11]. Combined, these studies show that CAs are important therapeutic targets for the treatment of an expanding array of human diseases.

Recent advances in the field of CA research show that they may also play an important role in the process of tumorigenesis, cancer progression, and metastasis [10–20]. It has become clear that specific isoforms (CA IX and CA XII) are more commonly overexpressed in tumor cells in comparison to normal healthy tissues [12, 15, 21–24]. These are termed the “tumor associated isoforms,” while others are more broadly expressed in normal cells including the blood [25]. This difference in expression pattern makes the design of isoform selective CA inhibitors, which specifically target the tumor-associated isoforms an important undertaking [26]. Acetazolamide, a sulfonamide and the first clinically approved CA targeting drug for the treatment of glaucoma, is not selective and targets most CA isoforms [27–29]. Yet, two other sulfonamide based, CA targeting, small molecule inhibitors (SLC-0111 and indisulam) are currently in phase II clinical trials for the treatment of solid tumors [30–33]. Additionally, several other inhibitors, including both small molecules and biologics, are being developed to specifically target the CA IX and CA XII [21, 31, 32, 34–41]. Increasing isoform specificity and selectivity will consequently increase treatment effectiveness and reduce cytotoxicity (offtarget effects) in patients [2, 26, 42].

This undertaking has, however, proven challenging because there are 12 catalytically active human CA isoforms, which are structurally homologous and share high sequence identity [26, 43]. Additionally, most of the conserved residues are located within the active site [26]. At the core of the human CA active site, a zinc metal is tetrahedrally coordinated to three imidazole rings from His 94, 96, and 119 (using CA II numbering) [44–46]. This coordinated zinc metal is essential for the catalytic conversion of carbon dioxide into bicarbonate and a proton in the presence of water, the physiological reaction catalyzed by CAs [25, 44]. The catalytic site is located in an ~15 Å conical cavity and is surrounded by both hydrophobic and hydrophilic regions that provide accessibility to solvent, carbon dioxide, bicarbonate, and inhibitors [26, 47].

Recently, a “selective pocket” was proposed to extend between 10 and 20 Å away from the catalytic zinc that may serve to aid in the design of CA isoform selective inhibitors [47]. This pocket, containing several amino acids that differ among the CA isoforms, is currently the focus for designing isoform specific inhibitors, using the extended tail approach [26]. Other approaches have also been adopted in the development of isoform specific inhibitors that interact in the less conserved regions, outside of the active site, to inhibit catalysis [25]. CAs also exhibit a slower esterase activity mediated by the same catalytic pocket with a mechanism similar to that of the hydratase/CA activity [48]. Although the role of this second activity in cancer is currently unknown, many investigators often use the esterase reaction as an indicator of hydratase activity [49].

**Figure 1:**
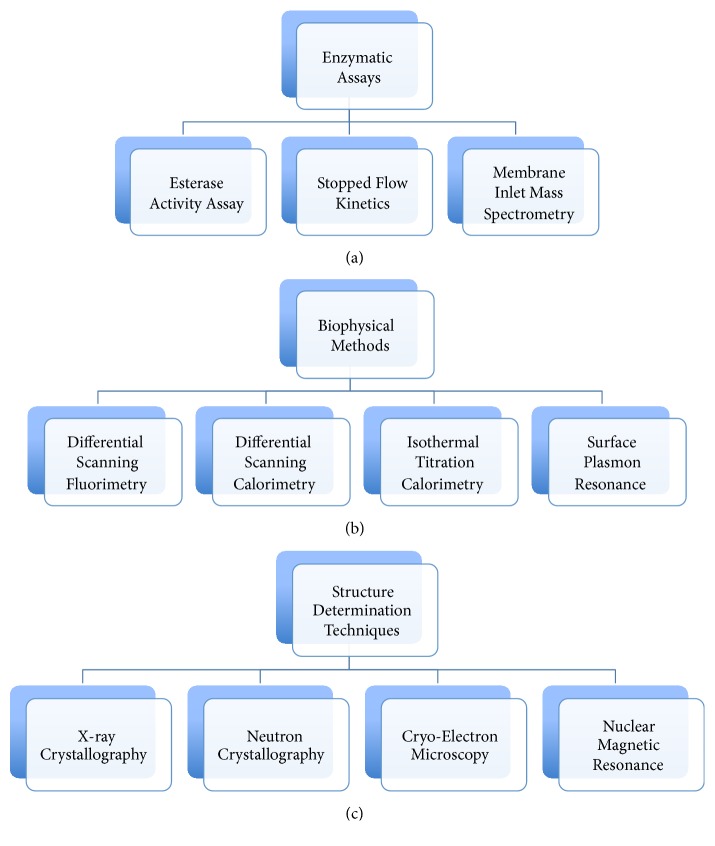
Biophysical and biochemical methods used to investigate the potency of CA targeted inhibitors, CA structure determination and drug development.

**Figure 2:**
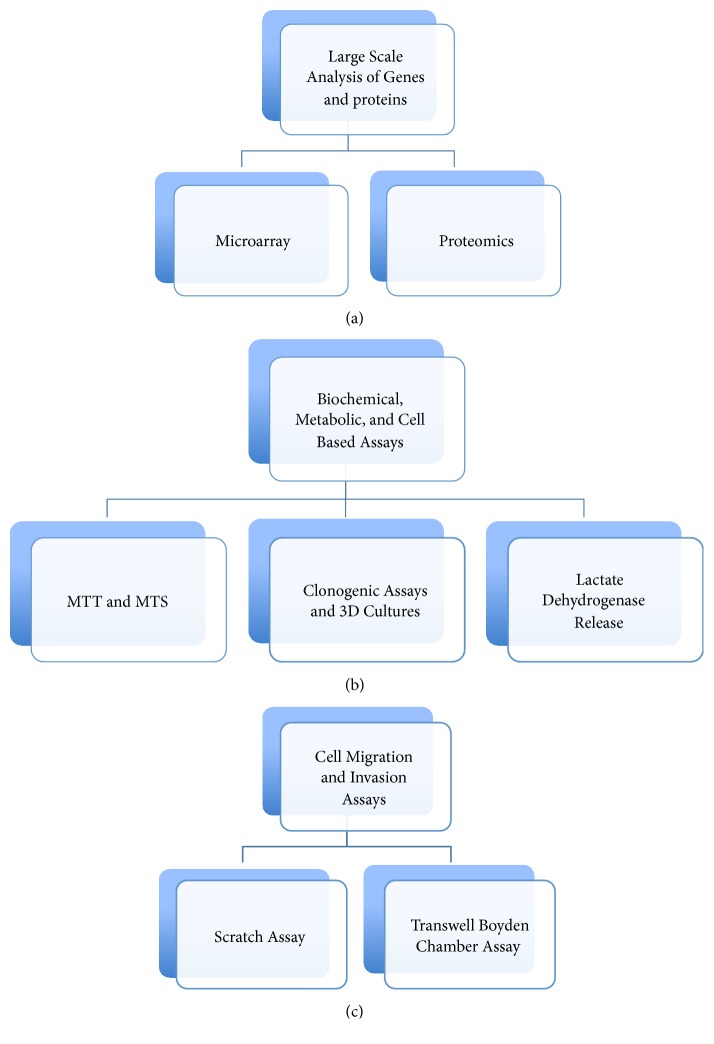
Large-scale analysis and cell based assays currently used to determine the role of CAs in cancer and the anticancer properties of CA targeted inhibitors.

A variety of compounds and biologics have been described in the literature that target different CA isoforms in many disease models. The potency, specificity, and efficacy of these inhibitors have been tested using different techniques and approaches. In this review, we focus on some of the biophysical, biochemical, and cell based approaches currently studied to evaluate the potency of CA targeted inhibitors and to decipher the role of CAs in cancer. Some of the approaches discussed include those that investigate inhibition of the CA enzymatic activity like esterase activity assays and stop flow kinetics and biophysical methods such as thermal shift assays, surface plasmon resonance, mass inlet mass spectroscopy (MIMS), X-ray crystallography, Cryo-EM, neutron crystallography, and nuclear magnetic resonance (NMR) (Figure 1). Other approaches that will be discussed include large-scale gene and protein expression, measure metabolism, pH regulation, cell growth, migration, and invasion (Figure 2). Together, these methods will hopefully reveal the role of CAs in tumorigenesis, cancer progression, and metastasis.

## 2. Enzymatic Assays and Biophysical Methods That Evaluate the Potency of CA Inhibitors

There are many techniques and strategies that have been described in the literature that evaluate CA activity for both purified versions of the enzyme and “intact” cells. These studies have been performed in the presence and/or absence of CA targeting inhibitors. Some of the techniques include measuring CA esterase activity on an ester substrate or the conversion of carbon dioxide into bicarbonate and a proton, which include stop flow kinetics and MIMS. Biophysical approaches such as differential scanning fluorimetry (DSF), differential scanning calorimetry (DSC), isothermal titration calorimetry (ITC), and surface plasmon resonance (SPR) also demonstrate binding of CA inhibitors to different isoforms. These techniques, however, do not indicate the regions of the proteins where inhibitors bind or their mode of inhibition (Figure 1).

Structural biology techniques such as those discussed in this review, X-ray crystallography, Cryo-EM, neutron crystallography, and NMR, have been used to determine CA structures, most often in complex with inhibitors (Figure 1). These strategies have provided information related to the structure of several CA isoforms and the interactions with CA targeted inhibitors. Supuran and coauthors have developed several CA inhibitors, and their modes of binding have been determined using one or more of the abovementioned techniques. These inhibitors can be subdivided into five groups: (1) compounds that interact directly with the catalytic zinc (sulfonamides and their isosteres, dithiocarbamates and their isosteres, hydroxamates, etc.); (2) compounds that anchor to the zinc bound water/hydroxide ion (phenols, polyamines, sulfocoumarins, etc.); (3) compounds that occlude the CA active site entrance (coumarins and their isosteres); (4) compounds that bind out of the active site region (carboxylic acid derivatives); and (5) compounds with unknown binding mechanisms (secondary/tertiary sulfonamides, etc.) [25].

## 3. Enzymatic Assays (Figure 1(a))

### 3.1. Esterase Activity Assay

The CA esterase activity, which is similar in mechanism to its hydratase activity, was first observed and measured in the mid-1900s [48]. In this study, the authors showed that the esterase activity of human isoforms is significantly lower than that observed for the hydratase activity [48]. However, like the CA hydratase activity, the CA targeted small molecule inhibitor, acetazolamide, also inhibits its esterase activity [48]. Since the discovery of this similarity in inhibition, numerous studies have relied on the measure of CA esterase activity as a surrogate for selecting CA inhibitors that block hydratase activity [49, 50]. In this regard, a commercially available, high-throughput colorimetric assay has been developed to determine CA activity in biological samples, serum, and purified CA (BioVision Incorporated). This assay utilizes the esterase activity of an active CA on an ester substrate, which releases a chromophore. The released product (4-nitrophenol) can be readily measured and quantified using a microplate reader (absorbance 405 nm). In the presence of a CA specific inhibitor, the enzyme's decrease in activity directly correlates to the decrease in absorbance. Currently, most of the CA related work using this technique has focused on purified recombinant proteins assaying only one CA at a time. Perhaps this assay can be optimized to measure CA activity in either cancer cells or lysates from different samples (both tumor and normal) although this might prove challenging as biological samples are much more complex often with more than one CA present. Assigning the activity to a specific CA isoform, in this setting, will be difficult. To overcome this potential issue, expression/concentration of the different isoforms in a sample would have to be known prior to running the assay.

### 3.2. Stopped Flow Kinetics

Stopped flow kinetics is a technique used for studying fast chemical reactions in solution. This spectrophotometer measures the fluorescence emitted when two molecules interact with each other. Its main advantages include small sample size and shorter experimental duration time. This technology can extend beyond enzymes to protein folding and catalysis reactions. It can also help determine rate constants and evaluate inhibition properties. The dynamics of this assay has been applied and optimized to evaluate the potency of CA targeted inhibitors, coined the stopped flow CO_2_ hydration assay (SFA) [51, 52]. Since the discovery of its ability to measure catalyzed CO_2_ hydration rate more than five decades ago, SFA has been widely used. The changes in absorbance of phenol red, a pH sensitive dye, is monitored for the activation or inhibition of CA catalyzed CO_2_ hydration reaction [51–53]. The half-maximal inhibitory concentration, IC_50_, is determined by fitting the dose response curves of the compound to the Hill model or the Morrison equation [54]. The inhibition constant, K_i_, is obtained from the IC_50_ using the Chen-Prusoff equation [55]. This technique has been widely applied by Supuran and coauthors to develop large libraries of CA targeted inhibitors, both small molecules and biologics, with diverse modes of binding and isoform selectivity [25]. The main limitation of this approach is that its use is limited to only simplified systems, as it is unable to discern the different CA activities in a more complex physiological setting or in altered environments and disease states.

### 3.3. Membrane Inlet Mass Spectrometry (MIMS)

**Figure 3:**
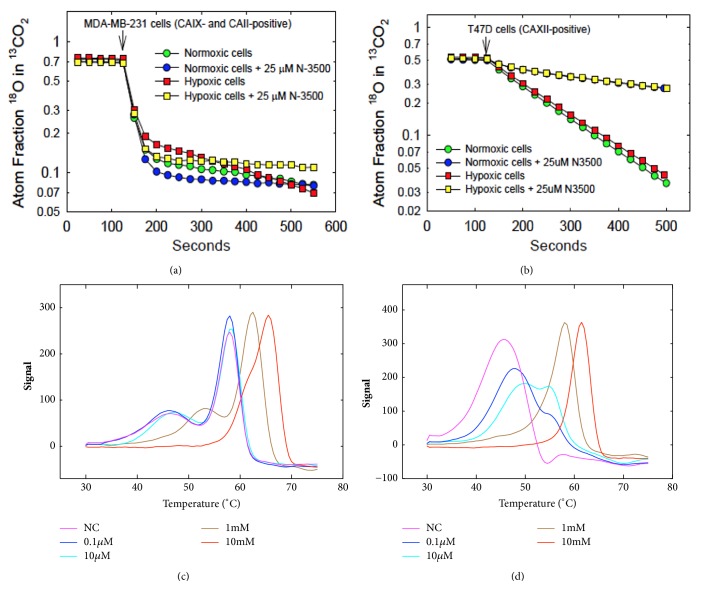
(a) CA IX and CA II and (b) CA XII activities were assessed using MIMS, under normoxic or hypoxic conditions for 16 h in the presence or absence of the impermeant sulfonamide inhibitor N-3500. DSF was used to determine the binding affinity of N-3500 to purified (c) CA II and (d) CA IX.

MIMS measures CA hydratase activity both in purified recombinant proteins and in the context of cancer cells (Figures 3(a) and 3(b)) [2, 56–59]. As such, this method is a direct approach in studying the physiological reaction of CAs. The CA kinetic rates are obtained by measuring the exchange of ^18^O from species of CO_2_ into water determined by MIMS [56, 60, 61]. Briefly, the reaction measures the continuous exchange among isotopic forms of ^18^O-labeled CO_2_ ([1/2 (47) + (49)] /[(45) + (47) + (49)] where the numbers in parentheses represent the peak heights of the corresponding CO_2_ masses that have diffused across a gas permeable membrane [59]. The membrane is submerged in the reaction solution and connected by glass tubing to a mass spectrometer (Extra EXM-200). The catalyzed exchange and uncatalyzed exchange of ^18^O between CO_2_ and water at chemical equilibrium are measured in an otherwise unbuffered solution at a concentration of 25 mM bicarbonate [59]. Usually the reaction solution is maintained at 25°C for purified proteins and the ionic strength normalized at 0.2 M by adding Na_2_SO_4_ [2]. For experiments using cell preparations of both normal and cancer cells, the reaction temperature is maintained at 16°C [59]. At this lower temperature, the enzymatic reaction is slowed to better contrast the intracellular and extracellular CA activities. This temperature also prevents endocytotic events during the course of the experiment, which is accomplished at temperatures slightly below the phase transition for lipids in the plasma membrane. In the first of two independent stages of catalysis, the dehydration of labeled bicarbonate has a probability of transiently labeling the CA active site with ^18^O. In the second stage, the protonation of the zinc bound ^18^O-labeled hydroxide releases a species of H_2_^18^O, which is then released into solution and infinitely diluted. This approach has been successfully used to obtain rates catalyzed by CA in studies using purified recombinant CA IX and cancer cells (Figures 3(a) and 3(b)) [57, 59]. Although not a perfect system this method has been used to determine CA activity in the context of cancer cells, which sometimes express more than one isoform. This is particularly useful for discerning between a membrane-bound activity (like CA IX) and a cytosolic CA isoform (Figure 3(a)). However, its limitations should be taken into consideration when a specific cell line has two CA isoforms (or more) that have the same subcellular localization.

## 4. Biophysical Methods (Figure 1(b))

### 4.1. Differential Scanning Fluorimetry (DSF)/Fluorescent Thermal Shift Assay

DSF is known for its precision, simplicity, rapidity, and low requirement for protein concentration. DSF is used to measure activity between enzymes and their inhibitors (Figures 3(c) and 3(d)), obtain binding affinity data, and establish theoretical and experimental dose response curves [62–65]. This assay measures the shift in melting points of a ligand receptor complex and indirectly determines the affinity of a ligand for its protein target (Figures 3(c) and 3(d)). A protein's stability is related to its Gibb's free energy of unfolding, which is temperature dependent [64]. The probes used are environmentally sensitive and emit fluorescence when bound to the hydrophobic core of unfolded proteins [64]. DSF is ideally suited as a high-throughput screening method to efficiently evaluate the affinity of inhibitors to a target and to determine conditions that stabilize a protein. Ligands perturb the thermal stability of a protein upon binding [66]. This technique is also widely used in the CA field to investigate interactions between CA isoforms and various inhibitors and to determine thermal stability profiles of various isoforms (Figures 3(c) and 3(d)). For instance, the binding affinity of sulfamate and sulfonamide derivatives for CA II was obtained using this method in 2006 [67]. Additionally, DSF has been used to investigate the interactions between CA isoforms and benzene sulfonamide based inhibitors as well as saccharin sulfonamides [68–70]. The thermal stability profiles of recombinant human CA VB, CA VI, CA IX, and CA XII were also determined using this technique [71–74]. The major drawback to this technique is the fact that it is an indirect measure of binding and the probes used may interfere with components of the assay, which could lead to inconclusive results. For example, if a protein is extremely hydrophilic then the probe will not bind to most of the residues that make up the protein as it only interacts with hydrophobic residues. This will, therefore, affect the intensity of fluorescence emitted. If the signal is too low, the lower limit of detection of the instrument will be reached and the data provided will be inaccurate. If the inhibitors used are particularly hydrophobic then the probe might also interact more readily with the inhibitors than the proteins and that again will provide erroneous results.

### 4.2. Differential Scanning Calorimetry (DSC)

Watson and O'Neill developed DSC in the 1960s to study energy phase transitions through the change in enthalpy of biological compounds [75]. It is the most commonly used method to evaluate protein folding and stability (enthalpy of unfolding) [76]. This approach can provide an understanding of the stabilization of proteins through thermal denaturation. Determination of the enthalpy of unfolding measured by heating the protein solution at a constant pH. During a DSC experiment run, the difference in the amount of heat required to increase the temperature of a sample and reference is measured as a function of temperature (where the sample and reference are maintained at nearly the same temperature throughout the course of the experiment) [76]. The heat capacity of the reference sample is usually well defined over the range of scanned temperatures because the sample holder temperature increases linearly as a function of time [76]. A limitation of this approach is its constraint to temperatures above physiological relevance; i.e., these assay temperatures are higher than 37°C which is the temperature of most solid tumors. Therefore, the interpretation of the data is often extrapolated to better reflect biological conditions.

In the field of CA research, this technique has been used in combination with ITC, reviewed below, to determine the thermodynamics of CA unfolding and the increase in thermodynamic stability of CAs upon ligand binding [77, 78]. Results from some of these studies showed that the combination of DSC and ITC techniques give the dependence of the enthalpy of unfolding on a wider temperature range, an important condition to determine the nonlinearity of the enthalpy dependence in temperature. Furthermore, using DSC to confirm DSF data provides a higher level of accuracy in determining the melting temperature of enzymes. This combination was used by Mahon et al. to show that CA IX is more thermodynamically stable than CA II at low pH [2]. In another study, the binding affinity of several sulfonamides to CA isoforms (human CA I and bovine CA II) resulted in a good correlation in the rank ordering of binding affinity when analyzed by a combination of DSC and ITC [77]. This suggests that the combination of approaches provides a more detailed and accurate picture of the linkage between ligand binding and protein stability. Hence, DSF is a general tool to measure affinity, as ligands have effects on protein stability.

### 4.3. Isothermal Titration Calorimetry (ITC)

Since its invention and modification for biological applications, ITC has been used to directly measure the heat absorbed or released by a reaction and has become the method of choice for the study of protein-ligand interactions [79–82]. A single ITC experiment can provide information on binding constant, Gibbs free energy, enthalpy, and entropy of a system [83]. It can also determine ligand receptor interactions and binding affinities for different macromolecules. This method does not require modification of the target protein (epitope tagging or otherwise labeled) or immobilization [83]. Its disadvantages are that it requires large amounts of purified protein and is expensive and time consuming. That said, ITC is used to determine the enthalpy of unfolding by titrating acid at a constant temperature. It is also used to assess the energetics of intermolecular reactions, i.e., drug-target interactions. This helps to better understand the catalytic mechanisms and biochemical pathways. ITC is a label-free, highly sensitive, and automated technique that provides precise data on stoichiometry, kinetics, and thermodynamics in a single experiment [83–86].

This experimental approach requires that all the molecules of interest are in homogenous solutions and applicable for water-soluble systems. In addition, ITC can be performed with unmodified, native forms of macromolecules. In the current commercial titration calorimeters, the inhibitor solution from the syringe is injected at constant temperature into the protein solution, preloaded into the calorimeter cell, until all binding sites of the protein become occupied by the ligand. Numerous studies of CA isoform interactions with diverse ligands have been performed using ITC [78, 87–90]. Some of these studies include binding of inhibitors of specific CA isoforms, which is important for a better understanding of structure-activity relationships. Additionally, ITC standard and displacement titrations, when used in combination with other structure determination techniques such as X-ray crystallography, can provide information on the intrinsic, buffer independent binding affinity of compounds to human CA isoforms [2, 91].

### 4.4. Surface Plasmon Resonance (SPR)

SPR, first applied to monitor biomolecular interactions in 1983 by Lundstrom, uses a microchip that has an immobilized ligand or receptor on a thin piece of metal film [92]. The microfluidic channels, above the chip, flow solutions containing ligand over the chip. When polarized light is aimed at the metal film, a dip in reflected light occurs as photons are transformed into plasmons (a quantum of plasma oscillations). SPR can be used to determine kinetic constants, molecular weights, and binding affinities. It is label-free, performed in real time, and can be used for high-throughput screening. The variable measured is in resonance units (RU). In the last decades, SPR biosensors have become the state-of-the-art technology in diagnostics and biomedical research to determine real-time kinetics and binding affinities of ligand-protein interactions [93]. This discovery is not lost in the CA research field, as SPR has been used in many CA focused studies for screening of inhibitors, including those targeting the tumor-associated isoforms CA IX and XII [94–97].

## 5. Structure Determination Techniques (Figure 1(c))

### 5.1. X-Ray Crystallography

X-ray crystallography remains the most effective way to determine atomic models of proteins and nucleic acids [98]. It has become invaluable in the field of targeted drug design and for the determination of atomic and molecular structure of protein crystals [99]. The diffracted data can be reduced and summated to produce a 3D image of the electron density and determine the position of atoms within the crystal [45]. Other information such as chemical bonds, their disorder, and ligand bound interactions can also be obtained [45]. Programs like COOT are used to help visualize the active site and assess the usefulness of ligands [100]. The earliest structures solved by this method were simple and marked by 1D symmetry. As computational and experimental methods have improved, it has become almost routine to deduce reliable atomic positions for macromolecules at subatomic resolutions. This is evidenced by the increase in high-resolution structures deposited to the protein data bank (PDB) [101].

**Figure 4:**
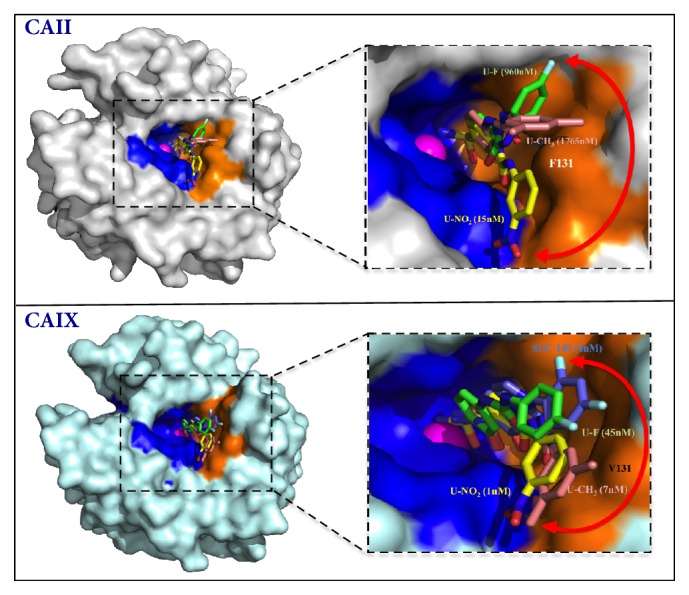
X-ray crystallography structures showing sulfonamide based inhibitors bound in the active site of CA II (top) and CA IX (bottom). Catalytic zinc (magenta sphere), hydrophilic (blue), and hydrophobic (orange) residues are as shown. Red double-headed arrows indicate isoform specificity relative to residue 131 (labeled in white). These arrows also show flexibility in tail conformations seen in CA II and CA IX. The K_i_ values of each compound bound to purified CA II and CA IX are also given. Figure was designed using PyMol.

The structures for most CA isoforms have been determined at high resolution, using X-ray crystallography methods, the coordinates of which are deposited to the PBD. These structures give detailed information about the 3D arrangement of residues within crystals of each isoform, while taking into account the residue differences among the isoforms (Figure 4). This has greatly advanced the CA drug development field because differences in amino acid residues within the CA active site can and have been explored, for the design and development of isoform selective inhibitors, especially for those that target the tumor-associated isoforms [26]. Furthermore, high-resolution structures of various inhibitors bound to several CA isoforms have also been determined. These inhibitor-bound structures provide structure function information and the mode of binding of each inhibitor (Figure 4). Major limitations to this method include the requirement of high concentrations of starting material in order to get crystals that can diffract to high resolutions [102]. Various other X-ray methods can be applied to obtain less detailed information, when material is limited. These methods include fiber diffraction, powder diffraction, small-angle X-ray scattering, and electron crystallography.

### 5.2. Neutron Crystallography

Although X-ray crystallography has been used to elucidate over 80,000 macromolecular structures, very few of them are well enough resolved to see individual H atoms [103]. Being able to visualize H atoms in a 3D context is important for understanding enzyme chemistry [104]. Therefore neutron crystallography represents a powerful tool for probing deeper into biological systems and revealing mechanistic information. One of the most powerful current applications of macromolecular neutron crystallography is determining protonation states of amino acid residues in the active site of enzymes as a means of addressing reaction mechanisms. A detained understanding of the water patterns and H-bonding in the active site provided by neutron diffraction provides a new avenue for rational structure-based drug design. In 2012, Fisher et al. published the first neutron crystallography structure of acetazolamide bound to the active site of CA II [105]. Although more than 500 X-ray crystal structures have been solved with this enzyme in complex with several inhibitors, these structures did not contain key details regarding H-atom positions of CA II and solvent and the charged state of the bond inhibitors was also missing [105].

Room temperature neutron structures of CA II in complex with sulfonamide inhibitors (acetazolamide and methazolamide) revealed details of hydration, H-bonding, and hydrophobic interactions between the inhibitors and CA II [106]. More importantly, these studies show that both inhibitors bound in their anionic forms. In recent years, an increasing number of neutron structures have been deposited in the PDB including enzyme-drug complexes. Although, the total number of neutron structures is still relatively small, there are a growing number of examples for which neutron crystallography has provided the answers to questions which remained elusive when other techniques were used. Neutron crystallography can also be used to determine the protonation states of several inhibitors and clinically used drugs for the treatment of cancer, bound to their targets under different conditions. For instance, changes in pH that occur within the tumor microenvironment may have a significant effect on the protonation states of inhibitors and may consequently affect binding to the targets and effectiveness.

### 5.3. Cryoelectron Microscopy (Cryo-EM)

Cryo-EM is a structure biology technique that is gaining popularity and is used to determine the 3D atomic coordinates of macromolecules [107]. It works by directing a beam of electrons at proteins that have been vitrified in solution. Further, Cryo-EM of frozen, hydrated samples does not require dehydration of the sample. It generates 2D images corresponding to a projection of the structure in the direction of the electron path. A near atomic resolution 3D reconstruction is obtained by combining images corresponding to different views referred to as “single particle” analysis [108]. Cryo-EM can be used to investigate a broad spectrum of drug-target interactions and dynamic conformational states [109–114]. As an alternative structure determination technique, some of its advantages include the fact that crystallization is not required and the sample size is small. To date, no CA structures have been solved using this method. This is because Cryo-EM enables the study of specimens larger than 150 kDa including viruses, small organelles, and macromolecular biological complexes as well as molecular interactions in supramolecular assemblies or machines [112–114]. The average molecular weight of CAs in the presence or absence of inhibitors is ~30 kDa [115]. However, this molecular weight should not deter structural biologists in the CA field from using this technique, especially, when studying CAs in complex with large biologics such as monoclonal antibodies. The mode of binding of most targeted antibodies to CA isoforms remains largely unconfirmed and unexplored due to limitations of the more popular X-ray crystallography techniques. Recent advances in the Cryo-EM field have the potential to change this, and soon CA-antibody complexes that provide information on structure relationships will be available.

### 5.4. Nuclear Magnetic Resonance (NMR)

NMR is a nuclei specific spectroscopy used to evaluate the structure and purity of samples both in solution and in solid state [116]. In this physical technique, the nuclei in a magnetic field absorb and re-emit electromagnetic radiation. The energy emitted depends on the strength of the magnetic field and the property of the isotope of the atoms at specific resonance frequency. NMR is often used to study molecular physics, crystals, and noncrystalline materials and to characterize protein-ligand interactions [116]. It is also routinely used in advanced medical imaging techniques, which include magnetic resonance imagining (MRI) [117]. Although larger sample size is required, relative to that for mass spectrometry, NMR is nondestructive. Furthermore, NMR is one of the two major spectroscopic techniques used in metabolomics. Therefore, it can be used to generate a metabolic fingerprint from biological samples to obtain information about disease states [118–120]. The latter is an important application in the field of CA cancer research, as some CA isoforms have been hypothesized to play a role in the tumor cell metabolism. Information about the metabolic fingerprint of tumors that express certain CA isoforms can be determined in comparison to non-CA expressing tumors and/or normal controls. Multiple studies in which CAs complexed with specific targeted inhibitors have already been successfully performed using this technique [121–123].

## 6. Large-Scale Analysis of Genes and Proteins in CA Cancer Research (Figure 2(a))

Genomic and proteomic analyses have gained popularity over the past few decades (Figure 2). These techniques detect global changes in gene and/or protein expression and are used to determine differences between normal and diseased states. Many of the “omics” techniques used in large-scale analysis of gene and protein expression have been applied to CA research. The most popular method of choice to determine genomic and genetic changes is through microarrays. Techniques like Northern blot and reverse transcriptase-polymerase chain reaction only allow for the testing of a few subsets of genes per experiment. However, microarray or “global gene expression profiling” looks at orders of magnitude more genes and the analysis is not often supervised (limited set of genes chosen prior to analysis). There are multiple types of microarrays, which include DNA microarrays, protein microarrays, peptide microarrays, tissue microarray, cellular microarrays, chemical compound microarrays, antibody microarrays, glycan arrays, phenotype microarrays, and reverse phase protein microarrays. Commonly used mass spectrometry techniques can obtain proteomic data, which is the large-scale study of proteins. Proteomics is more complex than genomics because the genome of an organism is to a degree constant, whereas the proteome differs from cell to cell and from time to time. Proteomics also provides a different level of understanding than genomics such as information about posttranslational modifications, alternative splicing, protein complexes, and protein degradation rates. Both technologies have been used in the field of CA research to determine the diagnostic and functional roles of different isoforms in cancer, which will be further discussed in this review.

### 6.1. Microarray

Microarray is a large-scale, high-throughput, screening method that can be used for the analytical and functional characterization of DNA, RNA, and protein [124–126]. Some functional parameters that can be deduced include regions of DNA that control normal and diseased states (functional genomics), protein–protein binding, enzyme substrate relationships, and even biochemical activity [127]. Some analytical uses are antibody arrays that, for example, can reveal altered mRNA and protein expression during cancer cell development, progression and metastasis [58, 128–130]. Although this assay has relevance beyond basic science research, with even clinical applications, its main advantages are its small sample size, automation, and high sensitivity. Despite the fact that it is an* in vitro *assay, this method shows much promise to becoming an indispensable research and clinical diagnostic tool. Protein and mRNA microarrays have also aided in the identification of biomarkers of disease and infection, of single nucleotide polymorphisms (SNPs), and of genes and proteins associated with therapeutic resistance and drug discovery [131]. Some disadvantages are the time consuming nature in preparing/purifying large numbers of proteins and its expense, at this point in time.

**Figure 5:**
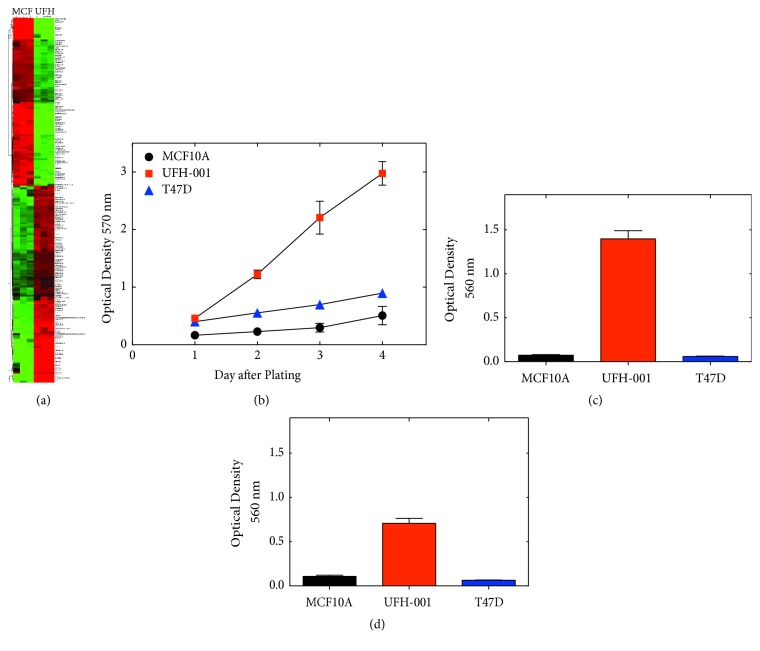
(a) Heatmap comparing microarray data of differentially expressed genes between UFH-001 (UFH, representative of triple negative breast cancer) and MCF10A (MCF, normal breast cells) cells (top 200 differentially expressed genes are shown). (b) Cell growth curves of MCF10A, UFH-001, and T47D (representative of luminal breast cancer) cells were determined using MTT assays. (c) Migration capacity of cell lines and (d) Invasion capacity of cell lines as determined using transwell Boyden chamber assays.

In CA research, comparison of gene expression levels between patients with different molecular subtypes showed differential expression of CA IX and XII ([132]. CA IX expression was mostly upregulated in patients with triple negative breast cancers and its increased expression was associated with decreased survival in these patients [58, 133]. Furthermore, Radvak et al. showed a significant decrease in the focal adhesion pathway upon CA IX depletion, through shRNA silencing in HT-1080 fibrosarcoma cells [20]. These data, obtained by microarray analysis, provided the first direct evidence for the role of CA IX in focal adhesion. Recently, published data by Chen et al. also showed that breast cancer cells that express high levels of endogenous CA IX correlate with a significant decrease in cell adhesion genes and increase in genes associated with metastasis, among many others (Figure 5(a)) [58]. Therefore microarray-based expression profiling allows for the identification of genes as well as the molecular and cellular events that might be important in complex processes. All of which are applicable in CA research and in deciphering the roles of CA isoforms in cancer.

### 6.2. Proteomics

The human genome contains approximately 20,300 genes. Proteomics is the global analysis of proteins, which are the key functional entities in the cell, required to understand how cells function [134]. Compared to the genome, the proteome has a higher order of complexity. Mass spectrometry based proteomics, which has developed considerably in recent years, has been become an invaluable tool to analyze the proteome [134]. These studies focus on posttranslational modifications, protein–protein interactions, and cellular localization. It is also used to annotate gene sequences and to find new protein-coding genes and splicing variants. The proteomic workflow begins with sample collection that often results in a set of samples from a diseased state, which is compared to appropriate controls. Mass spectrometry is then used to analyze the samples allowing for identification of large number of altered proteins [135].

The major difference between microarray and proteomics is the technologies used to obtain information and reproducibility [136]. For instance, the number of antibodies as well as their specificity to a particular protein limits the detection of changes in protein expression using microarrays. This problem is avoided when proteomic approaches are used. However, like microarray, proteomic approaches are expensive and the targets must be validated with analysis such as western blot and enzyme-linked immunosorbent assays (ELISA) [136]. Proteins identified in the initial cataloguing phase can be evaluated to provide further insights regarding their role in the pathogenesis of a given disease and their potential as a biomarker. Although very few studies in the CA field have used this technology, a recent paper published by Luo et al. identified CA II as a potential diagnostic biomarker for nasopharyngeal carcinoma [137]. This study was performed using the data independent mode based on sequential window acquisition of all theoretical mass spectra [137]. The metabolic interactome of CA IX determined using the proximity-dependent biotinylation proteomic approach, recently, revealed a novel role of this isoform in tumor migration and invasion [138].

## 7. Biochemical and Cell Based Assays to Decipher the Role of CAs in Cancer (Figure 2(b))

There are many biochemical and cell based techniques and strategies that have been described in the literature used to evaluate the efficacy of CA targeted inhibitors and decipher the role of CAs in cancer. These studies have been performed in the presence and/or absence of CA targeting inhibitors as well as using CA knockdown/knockout strategies. Some of the techniques commonly used include MTT and MTS assays, which measure mitochondrial function as an indicator of cellular viability and growth and lactate dehydrogenase (LDH) release assays that measure cytotoxicity (Figure 2). Assays that attempt to take the tumor microenvironment into account use the ability of cells to form colonies, such as 3D spheroids and clonogenic assays both of which are gaining popularity (Figure 2). 3D cultures are more representative of the* in vivo* environment than the more common 2D cultures. Taken together, the MTT/MTS, LDH, and clonogenic assays have been instrumental in evaluating the effectiveness of CA targeted inhibitors and effects of CA knock down/out on the growth, viability, cytotoxicity, and clonogenicity of cancer cells, all of which can be performed in 2D and/or the more physiologically relevant 3D culture systems. Finally, as large-scale genomic approaches have revealed a role of certain CA isoforms in metastasis, cell migration and invasion assays are now being used to confirm these findings on a functional level (Figure 2). The most commonly used techniques to evaluate cell migration and invasion include scratch/wound healing assay and transwell Boyden chamber assays. The scratch/wound healing assay is easier to use and inexpensive when compared to the Boyden chamber assays which are more accurate and reproducible. Together, these techniques have shown that membrane-bound CAs, especially isoform CA IX, are a key modulator of cancer metastasis.

### 7.1. MTT and MTS

The high-throughput, colorimetric tetrazolium salt-based assays, like MTT (3-(4,5-dimethylthiazol-2-yl)-2,5-diphenyltetrazolium bromide) and MTS (3-(4,5-dimethylthiazol-2-yl)-5-(3-carboxymethoxyphenyl)-2-(4-sulfophenyl)-2H-tetrazolium), are the most widely exploited approaches in cancer research for measuring cell proliferation, viability, and drug cytotoxicity [139, 140]. In living cells, the water-soluble yellow dye MTT is reduced to a dark purple (blue-magenta) colored formazan precipitate. The MTT formazan production is proportional to the number of metabolically viable cells. MTT reduction occurs throughout a cell and can be affected by a number of factors and substrates [141]. MTT is membrane permeable due to a net positive charge, whereas MTS has a net negative charge and largely cell-impermeable [139]. Moreover, MTS tetrazolium salts require an intermediate electron acceptor for reduction and form water-soluble formazans [139]. Combined, these two methods have been widely used to determine the difference in growth rates among different cell lines (Figure 5(b)) as well as the efficacy and anticancer effects of many CA targeting inhibitors. Results from studies in which these techniques were used collectively showed that most CA targeted inhibitors, specifically those that target the tumor-associated isoforms IX and XII, inhibited cancer growth. These effects were observed at high compound concentrations, with IC_50_ values >10 *μ*M [142–144]. The main limitation of this approach is that it does not provide direct evidence for cytotoxicity but it is often inferred [145]. MTT and MTS assays measure the metabolic rates of viable cells, which can often be inhibited by drugs. However, inhibition of cell growth is not indicative of cytotoxicity or cell death because cells can also become quiescence and the cells in the latter state are much more difficult to treat. To determine cytotoxicity, assays that specifically measure lactate dehydrogenase release, autophagy, and apoptosis may be used among others [146, 147].

### 7.2. Lactate Dehydrogenase Release (LDH)

LDH release assay is used to evaluate cellular cytotoxicity and cytolysis because LDH is released from the cytoplasm of damaged cells [146]. This colorimetric assay is ideal for high-throughput screening and it is also important because serum lactate dehydrogenase (S-LDH) is a well-known clinical surrogate parameter. A high activity of LDH is associated with poor prognosis in different tumor types. The released LDH can be quantified by a coupled enzymatic reaction. First, LDH catalyzes the conversion of lactate to pyruvate via reduction of NAD+ to NADH. Second, diaphorase uses NADH to reduce a tetrazolium salt (INT) to a red formazan product. Therefore, the level of formazan formation is directly proportional to the amount of released LDH in the medium. This assay is a better indicator of necrotic cell death and cytotoxicity [146, 148]. Recent studies in our laboratory show that some sulfonamide inhibitors affect cell growth without inducing cytotoxicity and vice versa (Mboge et al. submitted). It is, therefore, imperative that researchers are aware of the limitations of cell growth assays because they do not provide important information about drug cytotoxicity that LDH assays do.

### 7.3. Clonogenic Assays and 3D Spheroid Cultures

Clonogenic assays are commonly used for monitoring the efficacy of radiation modifying compounds and for determining the effects of cytotoxic agents and other anticancer therapeutics on the colony forming ability of different cell lines [149, 150]. The clonogenic assay enables an assessment of the differences in reproductive viability (capacity of cells to produce progeny; i.e., a single cell to form a colony of 50 or more cells) between control cells and treated cells. The assay also assesses the clonogenicity of cells that have undergone various treatments such as exposure to ionizing radiation, various chemical compounds (e.g., cytotoxic agents), or in other cases genetic manipulation [150]. 3D cell culture has garnered much interest in recent times. This system creates an artificial environment in which cells are allowed to grow and/or interact with their surroundings in all three dimensions. This 3D environment, unlike 2D cell culture, is more representative of how cells grow* in vivo*, which is usually in all directions. Earlier studies, led by Mina Bissell in the 1980s, highlighted the importance of 3D techniques for creating accurate* in vitro* culturing models. The focus of her work was on the importance of the extracellular matrix and the ability of cultures in artificial 3D matrices to produce physiologically relevant multicellular structures [151–154]. These include the formation of acinar structures in healthy and cancerous breast tissue models. Further, this technique has been applied to and for* in vitro* disease models that are used to evaluate cellular responses to pharmaceutical compounds [155–157]. Both techniques have been used to determine if CAs are important in clonogenicity and to determine how the tumor microenvironment affects the effectiveness of CA targeted inhibitors. For example, studies performed by Duivenvoorden et al. showed that shRNA-mediated knockdown of CA IX expression or pharmacological inhibition of its activity with sulfonamide based inhibitors significantly sensitized renal cell carcinoma (RCC) cells to ionizing radiation [158]. These experiments were performed using clonogenic survival assays and the results were recapitulated* in vivo* [158]. In the same year, the Yeger group also showed that CA II mediates malignant behavior of pulmonary neuroendocrine tumors and clonogenic assays were one of the techniques used [159].

## 8. Cell Migration and Invasion Assays (Figure 2(c))

### 8.1. Scratch/Wound Healing Assay

The scratch assay was the first method used to assess wound closure, in which a scratch is generated on a confluent cell monolayer [160]. The speed of wound closure and cell migration can be quantified by taking snapshot pictures with a regular inverted microscope at several time intervals [161]. The cell wound closure assay is one of the easiest, simplest, and inexpensive techniques to observe cell migration. When taken a step further, it can be used to observe morphological characteristics of an individual cell during migration. Following the analysis of wound closure many phenotypes may be revealed. Measuring the closed distance over time when comparing to a control may reveal specific migration changes or an impaired migratory phenotype that was previously unknown [160, 161]. Furthermore, single cell lamellipodium formation, tail retraction, and directional movement may give clues as to what may be impaired or enhanced in the cells of interest. This technique is used by many researchers, including researchers in the CA field, as an initial drug screening method to determine the effect of inhibitors on the migratory capacity of cells. It is often accompanied by other more stringent technologies such as transwell Boyden chamber assays to confirm initial observations. For instance, in 2013, Svastova et al. used both techniques to show that CA IX increases cancer cell migration via its catalytic domain [162].

### 8.2. Transwell Boyden Chamber Assays

The Boyden chamber assay is the most commonly used and most accurate technique to determine the migratory and invasive capacity of cells in culture [163]. It can also be used to analyze the ability of single cells to directly respond to various chemoattractants such as chemokines, growth factors, lipids, or nucleotides. Further, these assays identify and characterize the key regulators of cell migration and assess differential migratory ability of cells due to overexpression of a target or receptor. This technology is based on a transwell chamber system of two medium-filled compartments separated by a microporous membrane [164–168]. Cells are placed in the upper compartment and are allowed to migrate through the pores of the membrane into the lower compartment, in which chemotactic agents may be present. For cell invasion experiments, the insert, which separates the upper and lower compartments, is coated with an extracellular matrix (ECM) [163]. Cells are then allowed to break down the ECM (invade) in order to get to the lower compartment. Some advantages include its reliability, versatility in conducting motility experiments, and the relatively short duration of the experiment.

Many studies involving CAs have used this technology to determine the metastatic potential of cancer cells (Figures 5(c) and 5(d)) that express specific isoforms in the absence or presence of inhibitors or when function is blocked through knockdown strategies. For example, in 2010 Hsieh et al. showed the effects of CA XII expression on migration and invasion of breast cancer cells using Boyden chamber assays [14]. Their results showed that knockdown of CA XII in MDA-MB-231 cells significantly decreased cell migration and invasion by interfering with the activation of the p38 MAPK pathway [14]. Additionally, overexpression of CA XII rescued the observation. In 2011, it was demonstrated that CA IX increases tumor-associated cell migration and invasion, while an independent study performed by Radvak et al. showed that suppression of CA IX leads to aberrant focal adhesion and decreased invasion of tumor cells [19, 20]. Furthermore, studies performed in our lab showed that CA IX expression in UFH-001 cells (representative of aggressive triple negative breast cancer) but not CA XII expression on T47D (representative of luminal A breast cancers) contributes to the metastatic phenotype observed in the cells [58]. Collectively, these data indicate that the extracellular tumor microenvironment plays a big role in determining the metastatic potential of cancer cells that express different CA isoforms. Another example is the expression of CA II in hepatocellular cells carcinoma cells, which has been shown to inhibit the epithelial-mesenchymal transition and metastasis [169].

## 9. Conclusion

In this review, some of the biophysical, biochemical, and cell based techniques and/or approaches that are used to evaluate the potency of CA targeted inhibitors and to decipher the role of CAs in cancer are discussed. Techniques such as esterase activity assays, stop flow kinetics, and MIMs have been used to measure enzymatic activity of purified protein and in “intact” cells, in the presence or absence of inhibitors. X-ray crystallography, other structure determination techniques, and thermal shift assays have been used to determine the structure of CAs with or without inhibitors. These studies have helped inform the CA drug development efforts towards isoform selectivity for cancer treatment. Further, determining the effectiveness of inhibitors* in vitro* is also important for the development of drugs that target CAs in cancer. To determine this, researchers in the CA field have adopted large-scale genomic and proteomic analysis to determine the roles CAs in cancer, as well as cell growth, apoptosis, clonogenic, cell migration, and invasion assays to determine the efficacy of CA targeted inhibitors.

To date, only few CA targeted inhibitors have gotten to clinical trials for the treatment of cancer. Two examples are small molecule inhibitors (SLC-0111 and E7070- Indisulam) and another is a CA IX targeting monoclonal antibody (G250-Girentuximab) conjugated to a set of cytotoxic agents [30–33, 37–39, 170]. Specifically, SLC-0111 has been shown to be a potent inhibitor of CA IX activity using various biophysical and biochemical techniques [30, 31]. Its mode of binding, within the active site of CAs, was shown using X-ray crystallography, which also gave insight into its structure function relationship and selectivity towards CA IX [31]. Various studies have also shown that SLC-0111 inhibits cell growth and metastasis both* in vitro* and* in vivo* [170], which indicates its potential for use in humans. SLC-0111 recently completed phase I clinical trials in Canada (clinical trials.gov, NCT02215850) and is scheduled to start phase II for the treatment of solid tumors. Results from phase I studies showed that the drug was well tolerated with limited side effects. The techniques described in this review have been and will remain instrumental to the field of CA cancer research. However, the capabilities and limitations of each technique, as mentioned herein, must be taken into account when deciding which to use. Additionally, most CA inhibitors that work according to* in vitro *studies must also be tested* in vivo* using animal models including nonhuman primates before human testing.
